# *De Novo* Assembly of Mud Loach (*Misgurnus anguillicaudatus*) Skin Transcriptome to Identify Putative Genes Involved in Immunity and Epidermal Mucus Secretion

**DOI:** 10.1371/journal.pone.0056998

**Published:** 2013-02-20

**Authors:** Yong Long, Qing Li, Bolan Zhou, Guili Song, Tao Li, Zongbin Cui

**Affiliations:** 1 The Key Laboratory of Aquatic Biodiversity and Conservation, Institute of Hydrobiology, Chinese Academy of Sciences, Wuhan, Hubei, P. R. China; 2 University of the Chinese Academy of Sciences, Beijing, P. R. China; Auburn University, United States of America

## Abstract

Fish skin serves as the first line of defense against a wide variety of chemical, physical and biological stressors. Secretion of mucus is among the most prominent characteristics of fish skin and numerous innate immune factors have been identified in the epidermal mucus. However, molecular mechanisms underlying the mucus secretion and immune activities of fish skin remain largely unclear due to the lack of genomic and transcriptomic data for most economically important fish species. In this study, we characterized the skin transcriptome of mud loach using Illumia paired-end sequencing. A total of 40364 unigenes were assembled from 86.6 million (3.07 gigabases) filtered reads. The mean length, N50 size and maximum length of assembled transcripts were 387, 611 and 8670 bp, respectively. A total of 17336 (43.76%) unigenes were annotated by blast searches against the NCBI non-redundant protein database. Gene ontology mapping assigned a total of 108513 GO terms to 15369 (38.08%) unigenes. KEGG orthology mapping annotated 9337 (23.23%) unigenes. Among the identified KO categories, immune system is the largest category that contains various components of multiple immune pathways such as chemokine signaling, leukocyte transendothelial migration and T cell receptor signaling, suggesting the complexity of immune mechanisms in fish skin. As for mucin biosynthesis, 37 unigenes were mapped to 7 enzymes of the mucin type O-glycan biosynthesis pathway and 8 members of the polypeptide N-acetylgalactosaminyltransferase family were identified. Additionally, 38 unigenes were mapped to 23 factors of the SNARE interactions in vesicular transport pathway, indicating that the activity of this pathway is required for the processes of epidermal mucus storage and release. Moreover, 1754 simple sequence repeats (SSRs) were detected in 1564 unigenes and dinucleotide repeats represented the most abundant type. These findings have laid the foundation for further understanding the secretary processes and immune functions of loach skin mucus.

## Introduction

Fish skin has vital biological functions including chemical and physical protection, sensory activity, behavioral purposes, thermoregulation, hormone metabolism, maintenance of fluid balance and osmotic homeostasis [1], [2]. In general, fish skin is composed of three layers namely the epidermis, dermis and hypodermis [1], [2]. One of the most distinctive features of fish skin is the production of mucus by the unicellular glands of epidermis, mainly goblet cells and club cells [3], [4]. Compared with terrestrial vertebrates, fishes live in a more adverse environment which contains greater numbers of pathogenic organisms such as bacteria, fungi and parasites. As the interface between environment and the inner body, fish skin is persistently exposed to environmental stressors and provides an important first line of defense against the attachment and penetration of various invading pathogens [5]–[7].

The epidermal mucus is suggested to be one of the most important protective substances associated with fish skin [8]. This notion is supported by the isolation and identification of numerous immune factors in fish skin mucus, including immunoglobulins, antimicrobial peptides, lysozymes, protease, lectins, C-reactive protein and complement proteins [9]–[20]. Certain substances in fish mucus have been reported to function in promoting wound healing [8], [21]. Thus, the importance of fish skin mucus has attracted extensive research interests in recent years. However, due to the lack of genomic and transcriptomic data for non-model fish species, identification and characterization of bioactive substances produced by fish epidermis have been conducted using conventional biochemical methods, which restricts the ability of researchers to uncover the full repertoire of mucous substances. Therefore, molecular mechanisms underlying the development and maturation of mucous cells, the synthesis and release of mucus bioactive products, and the responses of mucus cells to environmental stressors and pathogens remain largely unknown.

Mud loach (*Misgurnus anguillicaudatus*) belongs to Actinopterygii Cypriniformes Cobitidae (http://www.fishbase.org), which is a freshwater fish widely spread in eastern Asia. The loach skin is composed of many mucous cells, which can produce a mucin rich mucus layer on the body surface. Mud loach is commercially important in China, both for traditional Chinese medicine and food purposes [22]. The cultured output of mud loach in China mainland has reached 204552 tons in 2010 [23]. A peptide prepared from the muscle [24] and a polysaccharide (named MAP) isolated from the skin mucus of mud loach exhibited anti-proliferative and apoptotic effects on human cancer cell lines [22], [25]. The polysaccharide MAP also displayed protective effects on immunological liver injury in mice [26]. Several antimicrobial peptides including misgurin [27], hepcidin [28] MAPP [29] were also identified from the whole body homogenates of mud loach. Despite the economical and medical significance of mud loach, limited genetic resources are currently available and only 22158 ESTs for this species can be found in public database.

The maturation of RNA sequencing (RNA-seq) technology and progresses in bioinformatics, especially the development of *de novo* assembly tools, provide a powerful platform for characterizing the transcriptome of various species. RNA-seq has been increasingly applied to a wide spectrum of non-model species. The transcriptome of several fish species including zebrafish (*Danio rerio*) [30], common carp (*Cyprinus carpio*) [31], silver carp (*Hypophthalmichthys molitrix*) [32], Asian seabass (*Lates calcarifer*) [33], guppy (*Poecilia reticulata*) [34], lake sturgeon (*Acipenser fulvescens*) [35], European eel (*Anguilla anguilla*) [36] and blunt snout bream (*Megalobrama amblycephala*) [37], has been characterized by RNA-seq. However, transcriptomic analysis of mud loach remains to be performed.

In this study, we aim to assemble and characterize the skin transcriptome of mud loach using RNA-seq. The sequencing was performed using Illumina Genome Analyzer IIx platform. The high quality reads were subjected to *de novo* assembly. The performances of assembly tools including Oases [38], Trinity [39] and SOAPdenovo-Trans (http://soap.genomics.org.cn/index.html) were compared and the data set produced by Oases were used for subsequent analyses. A total of 40364 unigenes (>100 bp) were finally assembled from the filtered short reads and the quality of the assembled transcriptome was confirmed by RT-PCR and Sanger sequencing. Blastx searches against the NCBI non-redundant (nr) protein database annotated 17336 sequences and 86.9% of these sequences exhibited high homology with fish proteins. Gene ontology and KEGG orthology annotations identified GO terms and KEGG pathways highly represented in the skin transcriptome of mud loach. The data presented here will lay the foundation for investigation of the molecular mechanisms underlying the immune functions of fish skin and the epidermis mucus production.

## Results

### Illumina paired-end sequencing and *de novo* assembly

**Figure 1:**
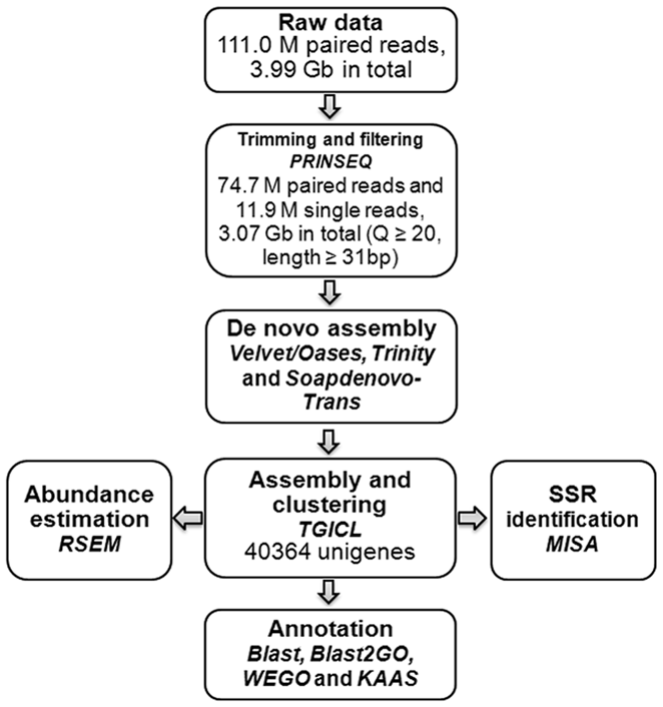
Flow chart of data analysis. The main steps and bioinformatics tools used for data analysis, volumes of raw and preprocessed data and number of assembled unigenes were displayed.

To characterize the skin transcriptome of mud loach, total RNA samples isolated from the skin of mud loach were subjected to library construction and high-throughput sequencing using the Illumina GA IIx platform. The main steps and bioinformatics tools used for data analysis were shown in Figure 1. A total of 111.0 M data for 36 bp paired-end reads (3.99 Gbp) were generated in this study. After trimming and filtering the raw reads by PRINSEQ with strict quality score threshold (Q≥20, length≥31 bp), 86.7 M of high quality reads (3.07 Gbp, 76.94%) were retained (Figure 1).

*De novo* assembly was performed using transcriptome assembly tools including Oases, Trinty and SOAPdenovo-Trans and performances of these tools were assessed according to N50 value, mean length, maximum length and transcript/scaffold numbers. Multiple k-mer values can be used by Oases and SOAPdenovo-Trans, while the version of Trinity used in this study takes a single k-mer value of 25. When compared to those from other two assembling tools at the same k-mer value of 25, the dataset generated by Trinity contained the highest number of transcripts and total bases, but exhibited the lowest continuity parameters (Table S1). SOAPdenovo-Trans gave higher continuity parameters including mean sequence length, maximum length and N50 value than Oases at all k-mer values; however, Oases used more reads and produced more transcripts. In addition, the transcripts assembled by Oases contained no gaps, but considerable portion of gaps (1.36–7.5%) were included in the assembly produced by SOAPdenovo-Trans (Table S1). As the optimal assembly of transcripts with different abundance requires different k-mer values, the combination of transcripts generated at different k-mer values would markedly improve the quality of assembly [40]. The merge of transcripts from different k-mer assemblies can be performed with Oases [38]. After the merging process, the contiguity parameters of the Oases assembly appear to be comparable with those of the best SOAPdenovo-Trans assembly (Table S1).

**Figure 2:**
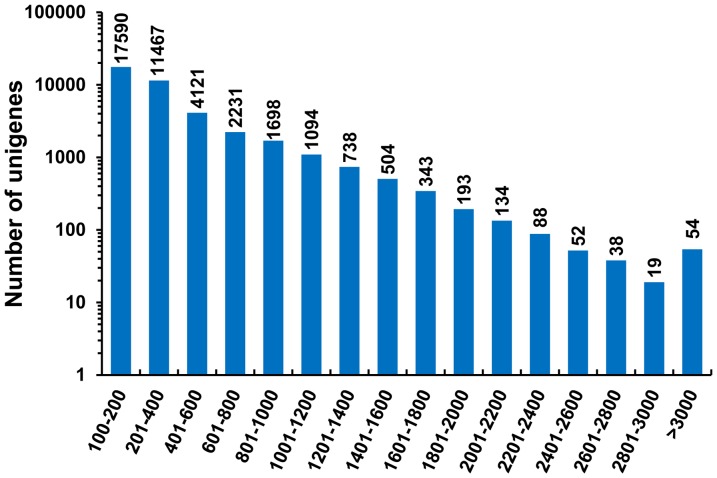
Length distribution of assembled unigenes.

Therefore, the merged transcript datasets from Oases assembly were used for subsequent analysis. The transcripts were further assembled and clustered using the TIGR Gene Indices clustering tools (TGICL) [41] with default parameters to reduce the data redundancy. The longest sequence in each cluster and the singletons were retained and designated as unigenes. Taken together, a total of 40364 unigenes were assembled. The mean length, N50 value and maximum length of assembled unigenes were 387, 611 and 8670 bp, respectively (Table 1). Length distribution of the assembled unigenes is displayed in Figure 2. The majority of sequences (71.99%) are ranged from 100 to 400 bp and 3257 (8.07%) unigenes are longer than 1 kb. These results are comparable with those of previous studies [42], [43].

**Table 1:** Statistics of the assembled transcripts and unigenes.

	Transcripts	Unigenes
Number of sequences	102010	40364
Total length (bp)	41247314	15626914
Mean length (bp)	404	387
N50 (bp)	588	611
Maximal length (bp)	8226	8670

### Quality assessment of the assembly

To evaluate the quality of the assembly, the unigenes were first aligned with the mRNA sequences of Misgurnus genus available in GenBank database using the blastn tool. Among the 57 mRNA sequences for *M. anguillicaudatus*, *M. mizolepis* and *M. fossilis*, 24 (42.11%) of them identified counterparts (e-values from 5E-70 to 0) in the mud loach skin transcriptome with sequence identities ranging from 88.35% to 99.85%. The missing of some sequences in our assembly may be attributed to the low abundance of these transcripts in the skin of mud loach. Additionally, more than 90% of the full length for 12 out of 17 mRNA sequences (70.59%) with complete CDS was found in the assembled skin transcriptome of mud loach (Table S2).

The sequence accuracy of assembled unigenes was further examined using RT-PCR and Sanger sequencing. A total of 25 unigenes with homology to known proteins were selected for this purpose. The primer sequences, size of amplicons, sequence description, length and abundance of these unigenes are displayed in Table S3. Total RNA samples isolated from skin, brain, gill, muscle, liver, intestine, testis and kidney were subjected to cDNA synthesis followed by RT-PCR analysis. Most of RT-PCR reactions at the first time produced specific amplicons, but nonspecific products were detected in reactions for two unigenes. However, these two unigenes were specifically amplified using redesigned primers, indicating that the previous unsuccessful amplification may be ascribed to the non-specificity of primers or assembly errors at the primer sites. The PCR products were sequenced and the sequencing data were aligned with the corresponding unigenes. As shown in Table S3, all the PCR products demonstrate the desired size and no insertion or deletion events exist in these sequences, but substitutions with an overall rate of 1.19% (199 out 16718) are found probably due to alternative splicing of exons or sequencing errors. Furthermore, most of these unigenes were extensively expressed in multiple tissues (Figure S1). These results indicate a high quality transcriptome of mud loach skin has been obtained through this study.

### Expression abundance of assembled unigenes

**Figure 3:**
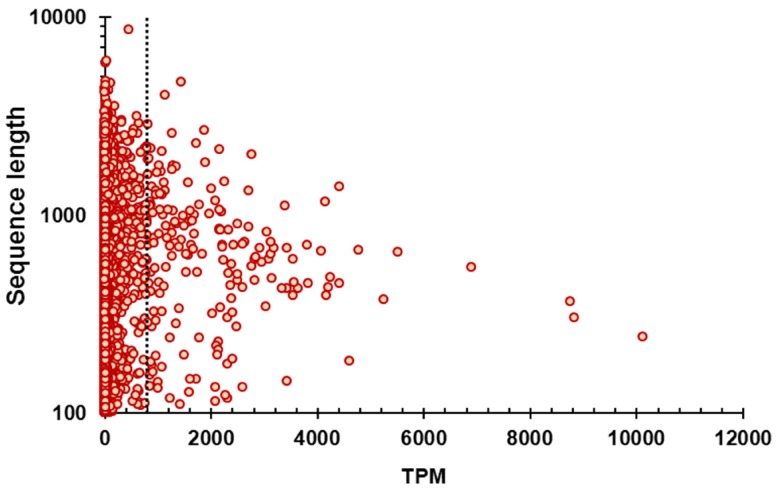
Relationship between the length and abundance of assembled unigenes. TPM: transcripts per million. The dashed line in the figure indicates that the abundances for most of the assembled unigenes were less than 400 TPM.

The abundance information is useful for understanding the function of identified genes. Relative abundance of the assembled unigenes was calculated using RSEM, which was reported to be accurate and powerful in abundance estimation of sequences from RNA-seq experiments, and was particularly suitable for *de novo* assemblies [44], [45]. When two mismatches are allowed in the mapping seed, a total of 68579975 (79.14%) reads were mapped back to the assembly with at least one alignment and the average sequencing coverage is 158-fold for the unigenes assembled in this study. The abundance of unigenes were expressed as TPM (Transcripts per million). This parameter is independent of the mean length of expressed transcripts and comparable across samples and species [44], [45]. The relationships between the length and abundance of unigenes are displayed in Figure 3 and most of the unigenes have an abundance value less than 400 TPM (Figure 3, Table S4). The top ten most abundant unigenes are displayed in Table 2 and the most abundant unigene was annotated as ictacalcin according to the result of blast search against the NCBI nr protein database (e-value = 1.83E-29). This gene encodes a calcium binding protein and was reported to be highly expressed in the skin of zebrafish embryos [46]. The rest of most abundant unigenes were annotated as keratin, ribosomal proteins, cytochrome c oxidase subunit, senescence-associated protein and ATP synthase subunit beta, respectively (Table 2). Obviously, these data will be of great value for further investigation of the physiological functions of fish skin.

**Table 2:** Top 10 most abundant unigenes.

Unigene ID	Abundance (TPM)	Length (bp)	Blast hit	Blast E-value
unigene9156	10117	242	ictacalcin	1.83E-29
unigene35922	8829	304	predicted: *Danio rerio* hypothetical loc795545	1.98E-16
unigene814	8754	363	predicted: *Danio rerio* keratin, type 1, gene 19d	1.43E-18
unigene36424	6898	542	*Cyprinus carpio* ribosomal protein L41	3.00E -117
unigene9284	5518	650	ribosomal protein S12	8.65E-86
unigene601	5254	374	cytochrome c oxidase subunit 1	9.07E-47
unigene455	4770	660	senescence-associated protein	3.72E-19
unigene35941	4600	182	*Danio rerio*, zgc:158463	2.93E-62
unigene2923	4417	452	60s ribosomal protein L35	3.73E-51
unigene802	4411	1384	ATP synthase subunit beta	8.63E-59

TPM: transcripts per million. The NCBI nr protein database, zebrafish transcriptome and NCBI nr nucleotide database were searched in sequential to annotate these unigenes.

### Functional annotation of assembled unigenes

**Figure 4:**
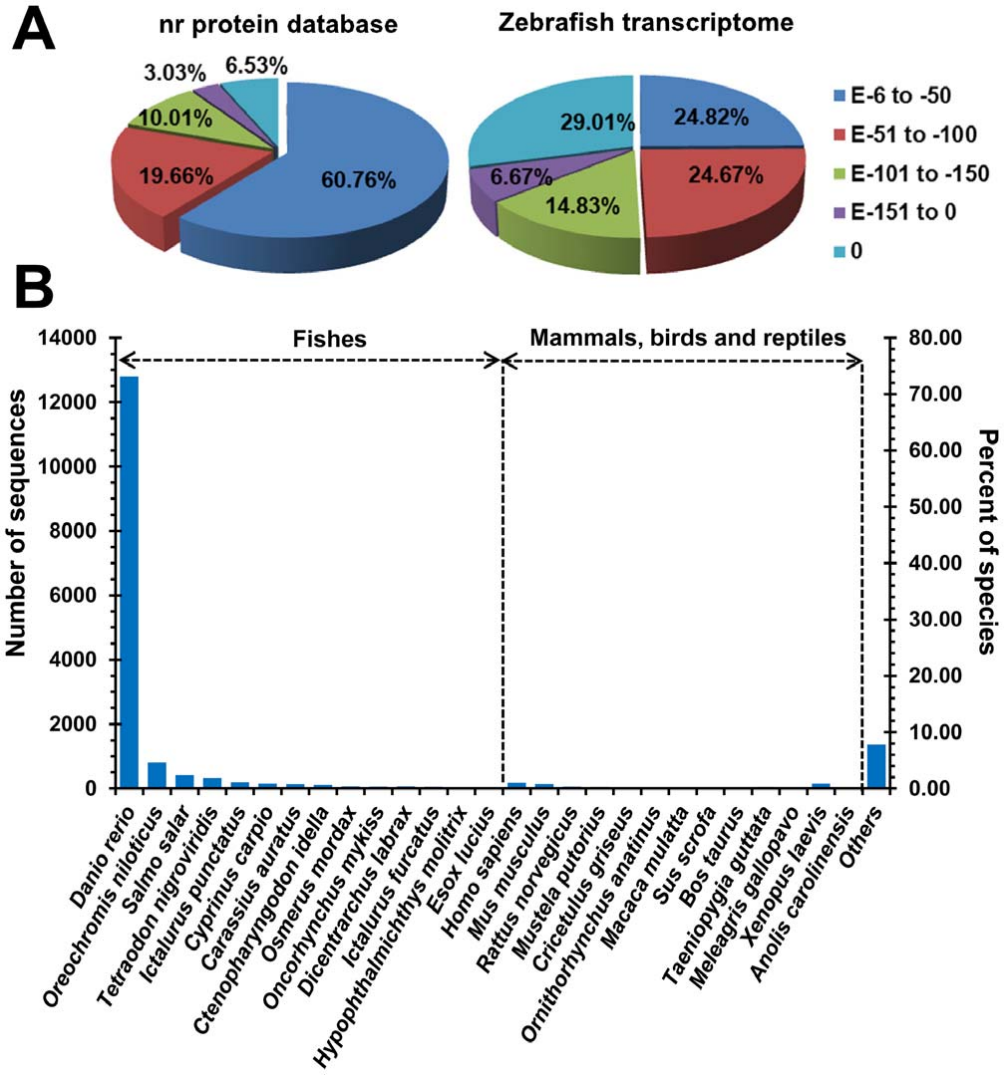
Blast e-value and distribution of top hit species. (A) Distribution of e-value for top hits obtained by blast searches of the unigenes against the NCBI nr protein database and zebrafish transcriptome. (B) Distribution of top hit species by blast searches against the NCBI nr protein database.

To further understand the mud loach skin transcriptome, the assembled unigenes were aligned to the NCBI nr protein database and zebrafish transcriptome. The number of unigenes with significant blast hits (e-value<1E-5) to known proteins and zebrafish transcripts were 17336 (43.76%) and 7615 (18.87%), respectively. The descriptions of best hits are shown in Table S4 and the e-value distributions of blast hits are displayed in Figure 4A. 6.53% of the blast hits against the nr protein database exhibited an excellent match (E-value = 0) and 60.76% of the sequences were annotated with blast E-values from E-6 to E-50, while these data were 29.01% and 24.82% for the blast hits from zebrafish transcriptome. These results suggest the relatively high homology of mud loach unigenes to zebrafish transcripts. The species distributions of best blast hits are shown in Figure 4B. 15062 (86.88%) blast hits against the nr protein database were originated from fish species and 779 (4.49%) hits were from other vertebrates including mammals, birds and reptiles. The remaining 1495 (8.62%) sequences were from other species mainly microorganisms. The relatively high ratio of blast hits from microorganism sequences may be ascribed to the attachment of microorganisms to the skin of mud loach and the high sequencing depth in this study.

**Figure 5:**
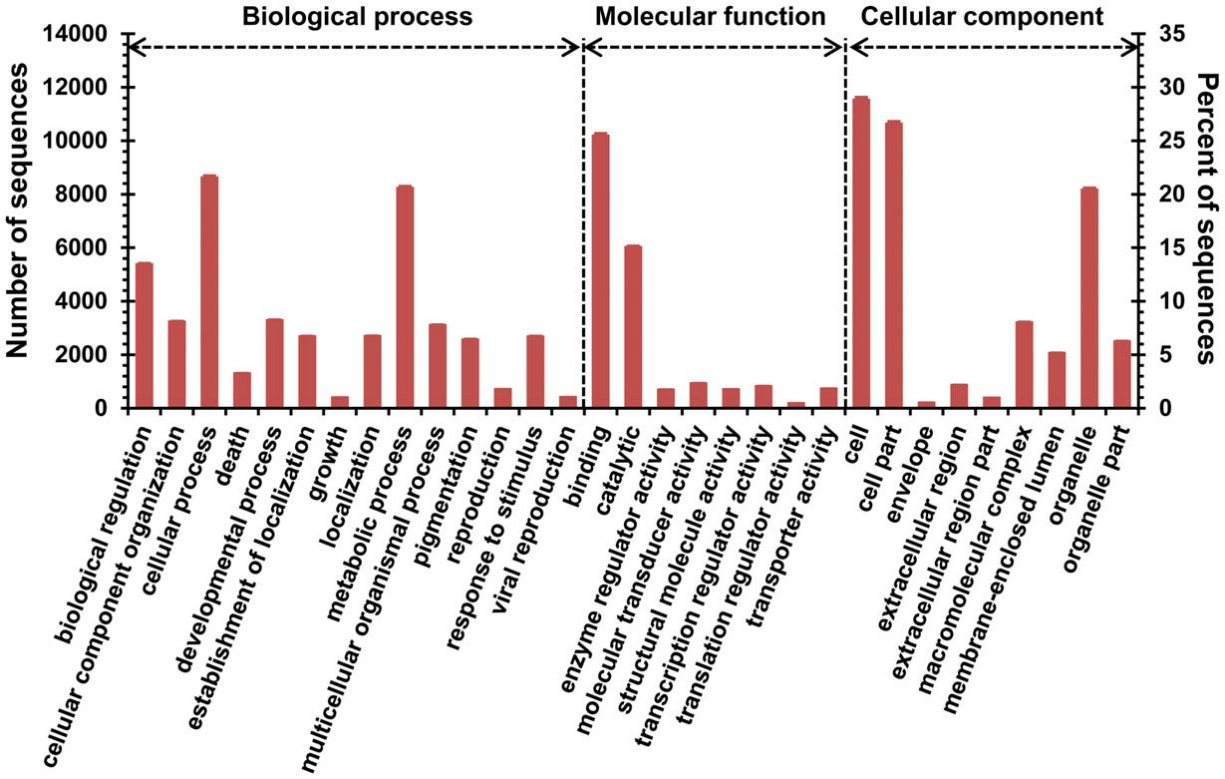
Gene ontology (GO) classification of assembled unigenes. Results of blastx searches against the NCBI nr protein database were imported into Blast2GO software for GO term mapping and annotation. Annotation results from Blast2GO analysis were submitted to the WEGO (http://wego.genomics.org.cn/cgi-bin/wego/index.pl) web server for GO classification. The number and ratio of sequences assigned to level 2 GO terms from sub GO categories including biological process, molecular function and cellular component were shown.

The results of blastx searches against the nr protein database were imported into Blast2GO [47] for GO mapping and annotation. A total of 108513 GO terms were assigned to 15369 unigenes (38.08% of the total assembled sequences). The numbers of mapped GO terms for biological process, molecular function and cellular component were 52231, 27565 and 28717, respectively. The GO classifications at level 2 of assembled unigenes are displayed in Figure 5. Cellular process (8748, 21.67%) and metabolic process (8355, 20.70%) were the most highly represented terms in the category of biological process, followed by biological regulation (5472, 13.56%), developmental process (3361, 8.33%), cellular component organization (3312, 8.21%), multicellular organismal process (3178, 7.87%), localization (2764, 6.85%), establishment of localization (2754, 6.82%), response to stimulus (2751, 6.82%) and pigmentation (2638, 6.54%). These data indicate that the genes expressed in mud loach skin are involved in a wide variety of biological processes. The most abundant molecular function ontologies were binding (10328, 25.59%) and catalytic (6122, 15.17%), whereas the remaining GO terms such as molecular transducer activity and transcription regulator activity were assigned to far less unigenes. In the cellular component category, cell (11679, 28.93%), cell part (10775, 26.69%) and organelle (8289, 20.54%) were the most abundant terms (Figure 5).

**Figure 6:**
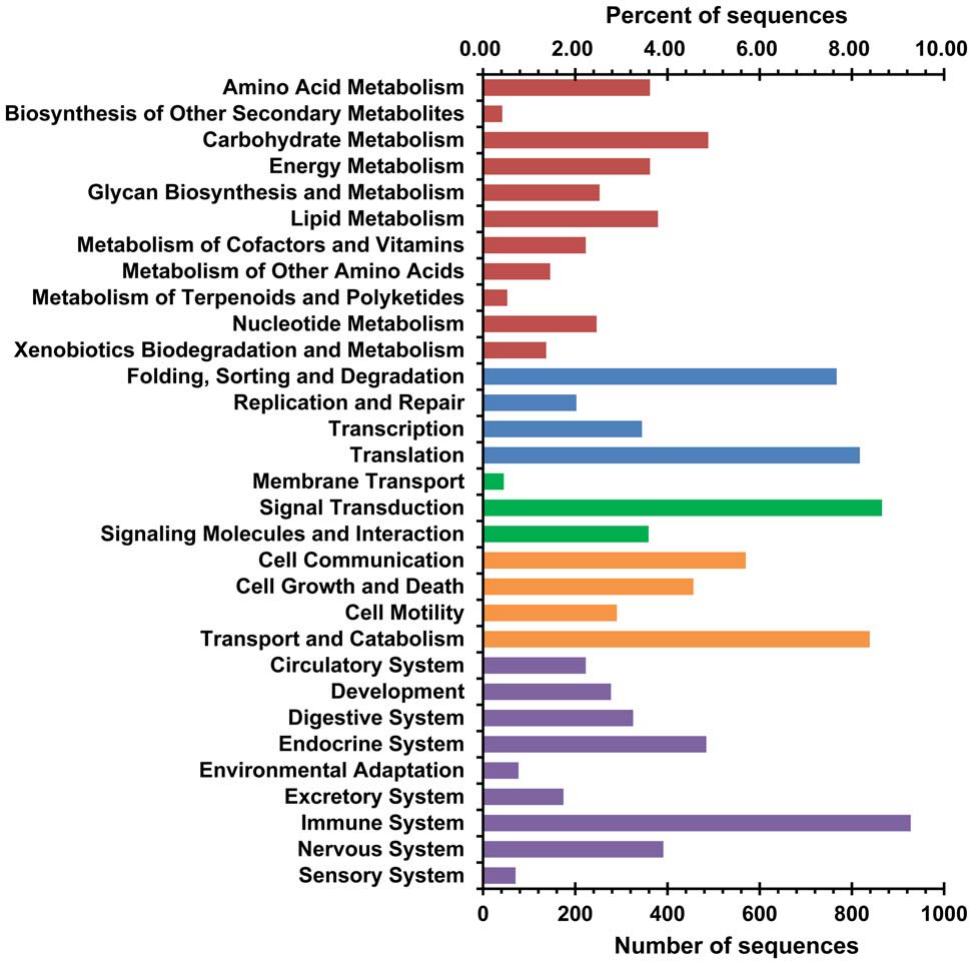
KEGG orthology (KO) classification of assembled unigenes. KO functional annotation was performed using KEGG Automatic Annotation Server (KAAS). The sequences of assembled unigenes were submitted to KAAS and the homology to KEGG genes were calculated using single-directional best hit (SBH) method. KO functional hierarchy of mapped pathways was viewed by KegHier software downloaded from the KEGG web site. The number and ratio of sequences assigned to each sub-category of the five top KO categories, namely metabolism (red), genetic information processing (blue), environmental information processing (green), cellular processes (organe) and organismal systems (purple), were calculated and displayed.

KEGG annotation of the assembled unigenes was performed using the KAAS [48]. A total of 9337 (23.23%) assembled unigenes were assigned with KO identifiers and 213 pathways were associated with more than 5 unigenes. The well represented pathways were focal adhesion (295), protein processing in endoplasmic reticulum (291), regulation of actin cytoskeleton (271), endocytosis (264), phagosome (264), RNA transport (257), MAPK signaling pathway (240), spliceosome (237) and ribosome (228) (Table S5). The number and ratio of sequences associated with each sub-category in the five top KO categories including metabolism, genetic information processing, environmental information processing, cellular processes and organismal systems, are shown in Figure 6. Among the identified functional categories, immune system (866), signal transduction (808), transport and catabolism (783), translation (763) and folding, sorting and degradation (716) were the most highly represented ones, followed by cell communication (532), carbohydrate metabolism (456), endocrine system (452) and cell growth and death (426). The results of GO and KEGG annotations provide firsthand information for investigation of the tissue-specific processes and functions of fish skin.

### Identification of non-coding RNAs

Given the existence of unigenes that were not mapped to known proteins and zebrafish transcripts by blast searches, we characterized the non-coding RNAs in the assembled dataset by searching against the Rfam database [49]. The Rfam database is a collection of RNA families, each represented by multiple sequence alignments, consensus secondary structures and covariance models (CMs) [49]. As shown in Table S6, a total of 138 unigenes without significant blast hits in the nr protein database were identified as putative non-coding RNAs (e-value<1E-3). Small nucleolar RNA and micro RNA were the most abundant ncRNA families with 26 identified members in each group and the number of associated unigenes was 27 and 30 (Table 3). Additionally, 33, 11, 6, 6, 3 and 3 unigenes were mapped to unaL2 LINE 3′ element, 5S ribosomal RNA, 5.8S ribosomal RNA, selenocysteine insertion sequence 1, tRNA and let-7 microRNA precursor, respectively (Table 3).

**Table 3:** Classification of identified non-coding RNAs.

NcRNA family	Number of NcRNAs identified	Number of associated unigenes
Small nucleolar RNA	26	27
microRNA	26	30
UnaL2 LINE 3′ element	1	33
5S ribosomal RNA	1	11
5.8S ribosomal RNA	1	6
Selenocysteine insertion sequence 1	1	6
tRNA	1	3
let-7 microRNA precursor	1	3

Only NcRNA families with more than 3 associated unigenes were shown.

### Immune pathways annotated from mud loach skin transcriptome

Although immune genes and pathways in fish tissues such as gill, liver, spleen, head kidney and larvae of turbot [50] and head kidney of grass carp [51] have been previously characterized using RNA-seq, the immune factors in the mud loach skin remains unknown. As shown in Figure 6, immune system was the most highly represented KO sub-category from the mud loach skin transcriptome, suggesting a large number of genes expressed in the skin are associated with immunity. All the immune pathways annotated in the mud loach skin transcriptome are listed in Table 4. Pathways associated with the highest number of unigenes were leukocyte transendothelial migration (216) and chemokine signaling (208), followed by Fc gamma R-mediated phagocytosis (145), T cell receptor signaling (141), antigen processing and presentation (139), natural killer cell mediated cytotoxicity (117) and Toll-like receptor signaling pathway (114). Among these immune pathways, Fc gamma R-mediated phagocytosis (42/55, 76.36%) and antigen processing and presentation (31/41, 75.61%) contained the highest ratios of identified genes versus the total number of known genes in the reference pathway. Thus, these results have provided an overview of the pathways involved in the immune functions of mud loach skin.

**Table 4:** Immune pathways annotated in the mud loach skin transcriptome.

Pathway name	KO identifier	Number of unigenes	Mapped genes	Known genes
Hematopoietic cell lineage	ko04640	29	18	78
Complement and coagulation cascades	ko04610	50	22	69
Toll-like receptor signaling pathway	ko04620	114	40	74
NOD-like receptor signaling pathway	ko04621	73	24	51
RIG-I-like receptor signaling pathway	ko04622	85	33	53
Cytosolic DNA-sensing pathway	ko04623	52	29	50
Natural killer cell mediated cytotoxicity	ko04650	117	44	79
Antigen processing and presentation	ko04612	139	31	41
T cell receptor signaling pathway	ko04660	141	55	81
B cell receptor signaling pathway	ko04662	91	37	52
Fc epsilon RI signaling pathway	ko04664	85	28	44
Fc gamma R-mediated phagocytosis	ko04666	145	42	55
Leukocyte transendothelial migration	ko04670	216	50	72
Intestinal immune network for IgA production	ko04672	55	12	37
Chemokine signaling pathway	ko04062	208	70	135

### Enzymes involved in the mucin type O-glycan biosynthesis pathway

Mucins are highly O-glycosylated glycoproteins ubiquitous in mucous secretions on cell surfaces [52]. Although the functions of mucins are well known and several fish mucin genes have been cloned [53], enzymes involved in the biosynthesis of mucins in the epidermal of fish remains to be characterized. A total of 37 unigenes from the mud loach skin transcriptome were mapped to 7 enzymes in the mucin type O-glycan biosynthesis pathway. The name of mapped enzymes, EC number and identity with corresponding enzyme from zebrafish are listed in Table 5. 24 unigenes assigned to the enzyme GALNT (EC2.4.1.41) were 82.86 to 98.59% homologous with the zebrafish N-acetylgalactosaminyltransferase family members including Galnt4, 5, 6, 7, 8, 11, 12 and 14. Other enzymes such as glycoprotein-N-acetylgalactosamine 3-beta-galactosyltransferase (EC2.4.1.122), beta-galactoside alpha-2, 3-sialyltransferase (sialyltransferase 4A, EC2.4.99.4) and C1GALT1-specific chaperone 1 (EC2.4.1.-) were mapped by 2 or 3 unigenes with sequence identity from 52.05% to 95.45%. These results provide interesting clues for the characterization of enzymes responsible for mucin biosynthesis in mucous cells of fish skin.

**Table 5:** Enzymes involved in mucin type O-glycan biosynthesis.

Enzyme	Symbol	EC number	Number of unigenes	Identity with corresponding enzymes in zebrafish (Accession#)
polypeptide N-acetylgalactosaminyltransferase	GALNT	2.4.1.41	24	
glycoprotein-N-acetylgalactosamine 3-beta-galactosyltransferase	C1GALT1	2.4.1.122	2	95% (NP_956345)
beta-galactoside alpha-2,3-sialyltransferase (sialyltransferase 4A)	SIAT4A	2.4.99.4	2	53% (XP_002664759)
beta-galactoside alpha-2,3-sialyltransferase (sialyltransferase 4B)	SIAT4B	2.4.99.4	3	52% (CAM56559.1)
C1GALT1-specific chaperone 1	C1GALT2	2.4.1.-	2	83% (NP_955961)
N-acetylglucosaminyltransferase 3, mucin type	GCNT3	2.4.1.-	2	80% (XP_002666963)
beta-1,6-N-acetylglucosaminyltransferase 4	GCNT4	2.4.1.-	2	79% (XP_001337774)

Notes: 1) At least two unigenes were mapped to each enzyme from zebrafish and the identity of the longest sequence with the enzyme was shown. 2) For GALNT, there were 24 unigenes mapped to 8 zebrafish enzymes; the sequence name, identity and accession number are as follow: Galnt4 precursor (91.91%, NP_001038243), predicted Galnt5-like (82.86%, XP_002667267), Galnt6 (86.35%, NP_998361), Galnt7 (95.91%, NP_001018477), Galnt8-like (88.64%, XP_697079), Galnt11 (90.68%, AAI24298), Galnt12 (82.88%, CAK05028) and Galnt14 (98.59%, NP_001038460).

### Genes associated with the SNARE interactions in vesicular transport pathway

**Figure 7:**
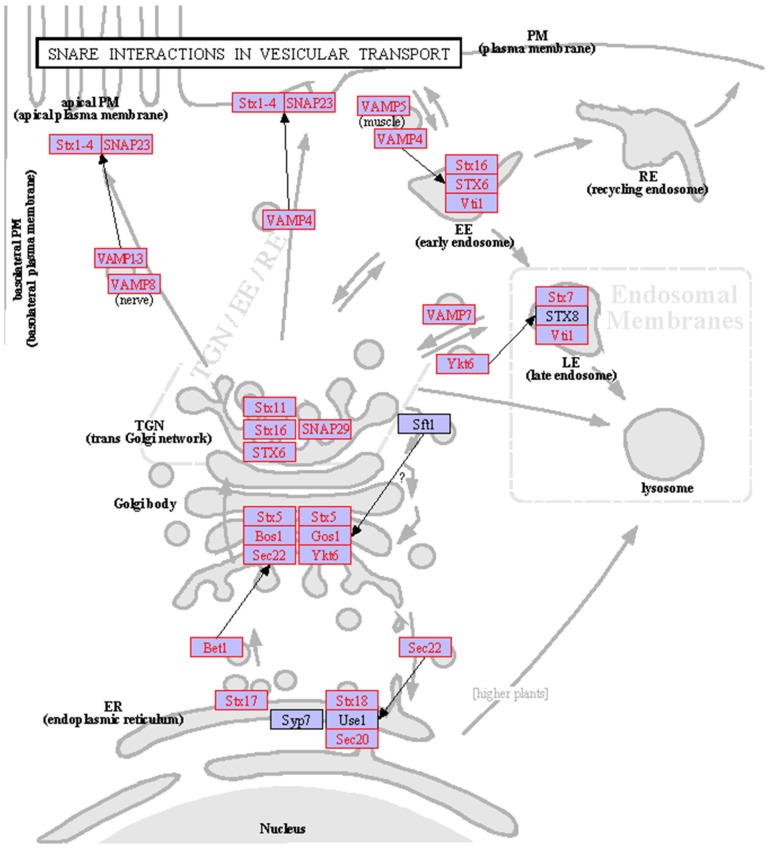
Map of the SNARE interactions in vesicular transport pathway. Genes identified from the transcriptome of mud loach skin were shown in red.

Like goblet cells in the airways and intestine of mammals, mucus secreting cells in fish epidermis package their products in secreting vesicles and release the contents through exocytosis [4], [54], [55]. Vesicle trafficking from endoplasmic reticulum to Golgi apparatus and then from Golgi apparatus to plasma membrane represent the main events during mucus secretion [55]. SNAREs are the key factors mediating membrane fusion between vesicles and their target membrane [56] and therefore play important roles in mucus secretion. In this study, a total of 38 unigenes were mapped to 23 genes of the SNARE interactions in vesicular transport pathway and only 4 factors in this pathway were not covered by the skin transcriptome of mud loach (Table S5 and Figure 7). The identified genes in this pathway included 9 syntaxins (Stx), 5 vesicle-associated membrane proteins (VAMP), 2 synaptosomal-associated proteins (SANP), 2 vesicle transport protein (Sec), 2 golgi SNAP receptor complex members (Gos1 and Bos1), Ykt6, Vti1 and Bet1 (Figure 7). These findings indicate the high activity of vesicular transport pathway in the epidermis of mud loach.

### Identification of EST-SSRs

**Figure 8:**
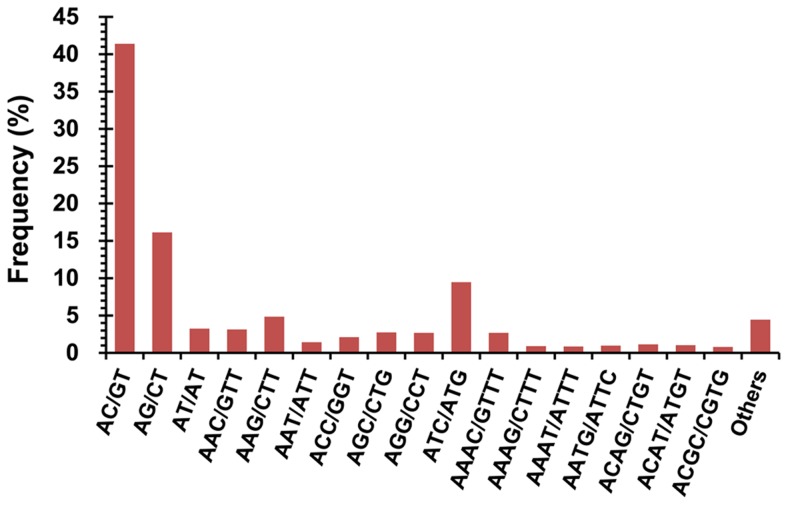
Frequency distribution of EST-SSRs based on motif types. A total of 162 EST-SSR motifs were identified and frequencies of main motif types were displayed.

Among various molecular markers, SSRs are highly polymorphic, easier to develop and very useful for researches such as genetic diversity assessment, comparative genomics and marker-assisted selection breeding [43]. To identify EST-SSRs, all the unigenes were searched using MISA. A total of 1754 EST-SSRs were detected in 1564 unigenes (3.87%) with a frequency of one EST-SSR per 8.90 kb sequence (Table 6). Among the identified EST-SSRs, di-nucleotide repeats represented the largest portion (60.78%), followed by tri- (27.08%) and tetra-nucleotide (11.00%) EST-SSRs, but only a small number of penta- (15) and hexa-nucleotide (5) EST-SSRs were identified (Table 6). Of the 162 EST-SSR motifs identified in this study, the numbers of di-, tri-, tetra-, penta- and hexa-nucleotide repeats were 10, 50, 85, 12 and 5, respectively. As shown in Figure 8, the AC/GT di-nucleotide repeat was the most abundant motif (726, 41.39%), followed by AG/CT (283, 16.13%), ATC/ATG (166, 9.46%), AAG/CTT (85, 4.85%) and AT/AT (57, 3.25%). These five types accounted for 75.09% of total motifs. The predicted EST-SSRs will be useful for charactering genetic diversity and marker-assisted selection breeding of mud loach.

**Table 6:** Statistics of EST-SSRs identified in mud loach skin transcriptome.

Searching item	Numbers
Total number of sequences examined	40364
Total size of examined sequences (bp)	15626914
Total number of identified SSRs	1754
Number of SSR containing sequences	1564
Number of sequences containing more than 1 SSR	169
Number of SSRs present in compound formation	104
Di-nucleotide	1066 (60.78%)
Tri-nucleotide	475 (27.08%)
Tetra-nucleotide	193 (11.00%)
Penta-nucleotide	15 (0.86%)
Hexa-nucleotide	5 (0.29%)

## Discussion

Fish skin plays various vital functions especially in immunity and defense against invading pathogens and environmental stressors [5], [57]. Recently, the immune functions of fish skin have attracted intensive interests of the research community, and a large number of antimicrobial and bioactive substances have been identified in the skin mucus of fish [15], [25], [58]. However, the molecular mechanisms underlying the biosynthesis, packaging and releasing of substances in skin mucus remain largely unknown due to the lack of genomic information such as the high quality skin transcriptome for economically important fish species. Mud loach, a freshwater fish with plentiful epidermal mucus on the body surface, has a relatively small body size and a strong ability of resistance to environmental stressors, and is amenable to experimental manipulations. Thus, mud loach has the potential to be a fish model for investigation of skin immunity and mucus secretion. Moreover, mud loach has long been used for food and medical purposes in eastern Asia. Therefore, characterization of its transcriptome will be of great value for the breeding, cultivation and disease prevention of this species.

In this study, we characterized the skin transcriptome of mud loach using Illumina paired-end RNA sequencing. Similar to the previous studies investigating the bark transcriptome of rubber tree [43] and the root transcriptome of sweet potato [59], this study has focused on the skin tissue and produced relatively short read length (36 bp), which is cost-effective and allows to increase the sequencing depth (111.0 M raw reads, 158-fold coverage) and strict read filtering thresholds. To improve the assembly, we compared the performances of three trancriptome assembly tools including Oases, Trinity and SOAPdenovo-trans. Oases was found to perform well than the other two tools in terms of used reads, assembled transcript numbers, total bases, continuity parameters and ratio of gaps. This may be ascribed to the combination of transcripts assembled with different k-mer values at the merging step of Oases since it has been previously reported that this approach can improve the performance of *de novo* assembly [40]. It is possible that Oases is more suitable for assembling relatively short reads. The assembly generated by Oases was thus used for subsequent analyses. After reducing the redundancy with TGICL clustering, a total of 40364 unigenes were finally assembled. The sequence accuracy of this assembly was independently confirmed by blast searches against available loach mRNA sequences deposited in GenBank database, RT-PCR and Sanger sequencing. The maximum length of assembled sequences is 8670 bp, indicating that extremely long transcripts are missing from the assembly; this may be attributed to the relatively short sequencing reads generated in this study.

A large number of unigenes were annotated by blast searches against the NCBI nr protein database (17336, 43.76%) and zebrafish transcriptome (7615, 18.87%), GO mapping (15369, 38.08%) and KEGG annotation (9337, 23.23%). The classification of GO categories including biological process, molecular function and cellular component provide overall information about biological functions of genes expressed in the skin of mud loach. The ratio of annotated sequences and the highly represented GO terms were comparable with previous studies performed with non-model species [32], [43]. The KO system provides a resource to link genomic data to higher-level systemic and functional information through KEGG PATHWAY and BRITE mappings [60]. The assembled unigenes were found to be involved in a wide array of KEGG pathways and KO categories. The predominantly represented pathways such as focal adhesion, regulation of actin cytoskeleton, endocytosis and phagosome all belong to the cellular processes of KO modules and are associated with molecular functions including cell communication, cell motility, cell proliferation, cell differentiation, intracellular transport and phagocytosis [61], [62], suggesting that multiple cellular events are active in the skin of fish. Other highly represented pathways like RNA transport, spliceosome, ribosome and protein processing in endoplasmic reticulum are involved in the gene expression processes including transcription, translation and protein folding, sorting and degradation. The activities of these biological processes may be the basis of the quick protein biosynthesis and secreting ability of fish epidermis.

The ability of fish to secret antimicrobial materials into skin mucus has recently attracted heavy research interests and a large array of antimicrobial peptides, lectins and antibacterial enzymes have been identified in fish epidermal mucus [6], [12], [18], [58]. However, the signaling pathways involved in fish skin immune responses remain to be defined. In this study, a total of 866 unigenes that represent the largest KO category were found to be associated with immune system (Figure 6). Multiple immune pathways were identified to be active in the skin of mud loach (Table 4). These pathways are known to be involved in various aspects of immune processes such as leukocyte transendothelial migration, immune signal transduction, phagocytosis, antigen processing and presentation. Moreover, many innate immune components such as lectins, lysozymes, cathepsins and complement factors were also identified in the mud loach skin transcriptome (Table S4). RNA-seq technology has been used to mine immune-related genes and pathways in several fish species [50], [51], [63]. Immune pathways including complement pathway, Toll-like receptor signaling pathway, B cell receptor signaling pathway, T cell receptor signaling pathway, apoptosis or programmed cell death and cytokines have been identified in the transcriptome generated using pooled RNA samples from gill, liver, spleen, head kidney and brain of turbot (*Scophthalmus maximus*), which was challenged with viruses [50]. In the spleen of large yellow croaker (*Pseudosciaena crocea*) infected with *Aeromonas hydrophila*, chemokine signaling, T cell receptor signaling, Leukocyte transendothelial migration and B cell receptor signaling pathway are found to be differentially regulated [63]. Findings from this study provide a full view of the immune components equipped with fish skin.

Mucins are the main constituents of fish skin mucus, which can form a matrix to trap and hold a wide range of antimicrobial molecules and are responsible for the gel-like nature of mucus. Mucins are high molecular weight glycoproteins characterized by their extensive O-glycosylation [52]. The O-glycosylation modification of mucins begins with the addition of N-acetylgalactosamine (GalNAc) to serine or threonine on the tandem repeats by activity of polypeptide N-acetylgalactosaminyltransferases [64]. The glycosyltransferases responsible for the elongation of O-glycan chain by sequential addition of carbohydrates are expressed in a cell- and tissue- specific manner [65]. The skin transcriptome of Atlantic salmon (*Salmo salar*) was recently assembled and several mucin genes were identified [66]. However, the enzymes involved in the biosynthesis of fish skin mucin remain not well known. In this study, 7 enzymes associated with mucin type O-glycan biosynthesis were identified from the mud loach skin transcriptome (Table 5). A total of 8 members in the polypeptide N-acetylgalactosaminyltransferases (EC2.4.1.41) family were found to be expressed in the skin of mud loach. These enzymes are active at the first step of O-glycosylation and belong to a large enzyme family containing at least 15 distinct members with tissue-specific expression and different substrate specificities [67]. The high redundancy of these enzymes in mud loach skin indicates the complexity of mucins produced by fish skin.

Similar to mucus secreting cells in the airways and intestine of mammals, goblet cells and club cells in fish epidermis package their products in secreting vesicles and release the contents through exocytosis [4], [55]. SNARE proteins are the key determinants that mediate the fusion of carrier vesicles with the target membrane during the vesicle traffic between cellular compartments [56]. In this study, nearly all the SNARE genes in vesicular transport pathway were identified from the mud loach skin transcriptome (Figure 7), suggesting the high activity of mucus secretion in mud loach epidermis. In the SNARE system, the vesicle membrane protein named v-SNARE interacts with the target membrane protein dubbed t-SNARE to form SNARE complex [56]. Among the identified SNARE molecules, the syntaxins (Stx), synaptosomal-associated proteins (SNAP) and Bet1 are t-SNAREs; while the vesicle-associated membrane proteins (VAMP), vesicle transport proteins (Sec), Ykt6 and Vti1 are v-SNAREs [68], [69]. The cell organelle specific distribution of these SNAREs accounts for the distinct vesicle transport events between different cellular compartments. Although all the key molecules in various vesicle traffic steps were suggested to be conserved from yeast to human [56], the regulation of mucus storage and release in fish epidermal mucus secreting cells may be quite different from the well characterized synaptic vesicle exocytosis. Thus, the results of this study provide valuable clues for charactering the responses of vesicular transport pathway in fish epidermis to various environmental stressors.

## Conclusions

In this study, we have assembled and characterized the skin transcriptome of mud loach using Illumina paired-end RNA sequencing. The assembling efforts generated 40364 unigenes. The sequence accuracy of this assembly was confirmed by RT-PCR and Sanger sequencing. A large portion (43.76%) of assembled unigenes was annotated by Blast searches. Functional classifications in terms of GO and KO have identified multiple biological processes and signaling pathways including those for immune systems and mucin biosynthesis in the skin of mud loach. To our knowledge, this is the first effort on assembling the transcriptome of mud loach skin. The data presented here will provide valuable resources for functional genomics of mud loach and investigation of mechanisms underlying the immune responses and mucus secretion of fish skin.

## Methods

### Ethics Statement

The animal protocol for this study was approved by the Institutional Animal Care and Use Committee of Institute of Hydrobiology (Approval ID: Y25E051501).

### Samples and RNA extraction

Mud loach (average body weight 15.42±2.63 g) were purchased from a local fishery market at Wuhan, China and acclimated to laboratory conditions for 2 weeks. The fish were anesthetized by placing into ice-slurry for 3 minutes and anesthesia was judged by loss of equilibrium [70], [71]. The fish were then euthanized by incising between the skull and the first cervical vertebra using a scalpel (cervical dislocation) [72]. Skin samples from the region between the dorsal fin and lateral line were collected with scissors and forceps. Total RNA was extracted with TRIZOL reagent from Invitrogen following the manufacturer's instructions. Total RNA contents were determined using the NanoDrop 8000 from Thermo Scientific. The quality of RNA samples was assessed by agarose gel electrophoresis. Equal volumes of total RNA from three individuals were combined and used for RNA-seq analysis.

### cDNA library construction and sequencing

cDNA library construction was performed by SinoGenoMax Co., Ltd, Beijing, China (http://www.sinogenomax.com/). Before library construction, the integrity of RNA samples was confirmed using Agilent 2100 Bioanalyzer and 4 µg of total RNA was used for isolation of polyA RNA with Sera-mag Magnetic Oligo (dT) beads from Illumina. The purified mRNA was fragmented into small pieces (100–400 bp) using divalent cations at 94°C for 5 minutes. Double-stranded cDNA was synthesized using the SuperScript Double-Stranded cDNA Synthesis kit (Invitrogen, Camarillo, CA) with random hexamer primers from Illumina. The synthesized cDNA was subjected to end-repair, phosphorylation, 3′ adenylation and adapter ligation in sequential. After these steps, cDNA fragments ranging from 250 to 350 bp were collected and purified by gel electrophoresis. Then, the purified cDNA template was enriched by PCR amplification and the quality of the library was validated in a LightCycler480 (Roche Diagnostics) using an Illumina PhiX174 Control. High throughput sequencing was performed by the Analytical & Testing Center at Institute of Hydrobiology, Chinese Academy of Sciences (http://www.ihb.ac.cn/fxcszx/). The constructed library was sequenced for 36 bp at both ends using an Illumina Genome Analyzer IIx platform according to the standard Illumina protocols as reported previously [59]. Sequencing experiments of constructed loach skin cDNA library generated a total of 111.0 M paired-end reads. The sequencing data have been deposited in NCBI Sequence Read Archive (SRA, http://www.ncbi.nlm.nih.gov/Traces/sra) with an accession number SRA057415. This Transcriptome Shotgun Assembly project has been deposited at DDBJ/EMBL/GenBank under the accession GAAD00000000.

### Data preprocessing and *de novo* assembly

The raw read data was preprocessed using PRINSEQ (version 0.19.3) [73]. The low quality (Q<20) and ambiguous bases (N) were first trimmed from both ends of the reads and then trimmed reads were filtered with Phred quality score (Q≥20 for all bases) and read length (≥31 bp). The trimming process spared reads with low quality bases at the ends. Paired reads were selected from preprocessed data sets using a perl script cmpfastq (http://compbio.brc.iop.kcl.ac.uk/software/cmpfastq.php) and a total of 74.7 M paired and 11.9 M single reads were obtained. The high quality reads were assembled using Velvet (version 1.2.07) followed by Oases (version 0.2.08) [38], [74], Trinity (r2012-06-08) [39] and SOAPdenovo-trans (release 1.01, http://soap.genomics.org.cn/index.html), respectively. The process of Oases assembling includes several steps [38]. The sequencing reads were first assembled using Velvet at distinct k-mer values (19, 21, 23, 25, 27, 29 and 31) and the contigs produced by Velvet at each k-mer value were further assembled into transcripts using Oases. Finally, the transcript data sets assembled at different k-mer values were merged using Oases with default settings (k-mer = 27). SOAPdenovo-Trans analysis was performed at the same k-mer values as with Velvet/Oases, while the k-mer value (k-mer = 25) was fixed in the version of Trinity used in this study. The performances of these assembly tools were assessed according to parameters including N50 value, mean length, maximum length and transcript/scaffold number. The data sets produced by Velvet/Oases were selected for subsequent analyses. To reduce data redundancy, the transcripts were assembled and clustered using TGICL [41] with default parameters. The longest sequence in each cluster and singletons were reserved and designated as unigenes.

### Quality assessment of the assembly

To assess the quality of sequences assembled in this study, the assembled unigenes were aligned to mRNA sequences of Misgurnus genus available in GenBank database using blast tools (version 2.2.26, http://www.ncbi.nlm.nih.gov/). Furthermore, 25 unigenes homologous to known proteins were validated by RT-PCR and Sanger sequencing. Total RNA samples were isolated from tissues including skin, brain, gill, muscle, liver, intestine, testes and kidney. cDNA template were synthesized using RevertAidTM First Strand cDNA Synthesis Kit from Fermentas. PCR primers were purchased from Sangon Biotech Co., Ltd., Shanghai, China. Specific PCR products were cloned and subjected to Sanger sequencing. The primer sequence, amplicon size and sequence description are displayed in Table S3.

### Abundance estimation

The relative abundance of assembled unigenes was calculated using RSEM (version 1.1.19) [45]. The unique feature of this tool is that it does not rely on the existence of a reference genome and it is therefore particularly useful for quantification with de novo transcriptome assemblies [45]. The preprocessed reads were first aligned to the reference generated from the assembled unigenes with single-end read mode. Then, the relative abundances of unigenes expressed as TPM were calculated from the alignment results.

### Functional annotation

The assembled unigenes were searched against the NCBI nr protein database (released on April 15, 2012 at http://www.ncbi.nlm.nih.gov) and zebrafish transcriptome (released on July 24, 2012 at http://www.ncbi.nlm.nih.gov) using blastx and blastn tools, respectively. E-value<1E-5 indicates the sequence conservation and the best aligning results were used to annotate the unigenes. The outputs of blast searching against the NCBI nr protein database were imported into Blast2GO program [47] for GO term mapping. The results of Blast2GO analysis were submitted to the WEGO [75] for GO classification under the biological process, molecular function and cellular component ontologies. KEGG annotation was performed using the single-directional best-hit (SBH) method in KAAS web server [48]. This tool is able to assign KEGG Orthology (KO) identifiers or K numbers to query sequences according to the sequence similarity and perform the pathway mapping and BRITE mapping processes [48]. The KO system is structured as a four level hierarchy. The top level consists of the following six categories: metabolism, genetic information processing, environmental information processing, cellular processes, organismal systems and human diseases. Each top level category contains a wide arrange of sub-categories (the second level). The third level corresponds directly to the KEGG pathways, and the fourth level consists of the leaf nodes representing the functional terms [60].

### Identification of non-coding RNAs and EST-SSRs

To identify putative non-coding RNAs from the assembled unigenes, sequences without significant blast hits in the nr protein database were searched against the Rfam non-coding RNA database using Rfam 11.0 (http://rfam.sanger.ac.uk/) [49]. Potential EST-SSR markers were detected within the assembled unigenes using the MISA microsatellite identification tool (http://pgrc.ipk-gatersleben.de/misa/). The parameters were adjusted for identification of perfect di-, tri-, tetra-, penta-, and hexanucleotide motifs with a minimum of 6, 5, 4, 4, and 4 repeats, respectively. Mononucleotide repeats were ignored since it is difficult to distinguish genuine mononucleotide repeats from the polyadenylation products.

## Supporting Information

Figure S1 **RT-PCR results for selected unigenes.**
(PDF)

Table S1 **Statistics of the assembly generated by Oases, Trinity or SOAPdenovo-Trans.**
(XLSX)

Table S2 **Quality assessment of the assembly by blastn analysis.**
(XLSX)

Table S3 **Unigenes selected for RT-PCR validation.**
(XLSX)

Table S4 **Length, abundance and description of the best blast hits of assembled unigenes.**
(XLSX)

Table S5 **Summary of unigenes involved in the KEGG pathways and KO categories.**
(XLSX)

Table S6 **Non-coding RNAs identified through searching against Rfam database.**
(XLSX)

## References

[1] Rakers S, Gebert M, Uppalapati S, Meyer W, Maderson P, et al (2010) ‘Fish matters’: the relevance of fish skin biology to investigative dermatology. Experimental Dermatology 19: 313–324. 20158518 10.1111/j.1600-0625.2009.01059.x

[2] Le Guellec D, Morvan-Dubois G, Sire JY (2004) Skin development in bony fish with particular emphasis on collagen deposition in the dermis of the zebrafish (Danio rerio). International Journal of Developmental Biology 48: 217–231. 15272388 10.1387/ijdb.15272388

[3] Harris JE, Hunt S (1975) The fine structure of the epidermis of two species of salmonid fish, the Atlantic salmon (Salmo salar l.) and the brown trout (Salmo trutta L.). I. General organization and filament-containing cells. Cell & Tissue Research 157: 553–565. 165897 10.1007/BF00222607

[4] Hawkes JW (1974) The structure of fish skin. I. General organization. Cell & Tissue Research 149: 147–158. 4424315 10.1007/BF00222270

[5] Raj VS, Fournier G, Rakus K, Ronsmans M, Ouyang P, et al (2011) Skin mucus of Cyprinus carpio inhibits cyprinid herpesvirus 3 binding to epidermal cells. Veterinary Research 42: 92. 21816061 10.1186/1297-9716-42-92 PMC3166907

[6] Ellis AE (2001) Innate host defense mechanisms of fish against viruses and bacteria. Developmental and Comparative Immunology 25: 827–839. 11602198 10.1016/s0145-305x(01)00038-6

[7] Palaksha KJ, Shin GW, Kim YR, Jung TS (2008) Evaluation of non-specific immune components from the skin mucus of olive flounder (Paralichthys olivaceus). Fish & Shellfish Immunology 24: 479–488. 18276162 10.1016/j.fsi.2008.01.005

[8] Shephard KL (1994) Functions for Fish Mucus. Reviews in Fish Biology and Fisheries 4: 401–429.

[9] Tsutsui S, Komatsu Y, Sugiura T, Araki K, Nakamura O (2011) A unique epidermal mucus lectin identified from catfish (Silurus asotus): first evidence of intelectin in fish skin slime. Journal of Biochemistry 150: 501–514. 21757471 10.1093/jb/mvr085

[10] Koo YS, Kim JM, Park IY, Yu BJ, Jang SA, et al (2008) Structure-activity relations of parasin I, a histone H2A-derived antimicrobial peptide. Peptides 29: 1102–1108. 18406495 10.1016/j.peptides.2008.02.019

[11] Shai Y, Fox J, Caratsch C, Shih YL, Edwards C, et al (1988) Sequencing and Synthesis of Pardaxin, a Polypeptide from the Red-Sea Moses Sole with Ionophore Activity. FEBS Letters 242: 161–166. 2462511 10.1016/0014-5793(88)81007-x

[12] Tasumi S, Ohira T, Kawazoe I, Suetake H, Suzuki Y, et al (2002) Primary structure and characteristics of a lectin from skin mucus of the Japanese eel Anguilla japonica. Journal of Biological Chemistry 277: 27305–27311. 11959866 10.1074/jbc.M202648200

[13] Aranishi F (1999) Possible role for cathepsins B and L in bacteriolysis by Japanese eel skin. Fish & Shellfish Immunology 9: 61–64.

[14] Park IY, Park CB, Kim MS, Kim SC (1998) Parasin I, an antimicrobial peptide derived from histone H2A in the catfish, Parasilurus asotus. FEBS Letters 437: 258–262. 9824303 10.1016/s0014-5793(98)01238-1

[15] Subramanian S, Ross NW, MacKinnon SL (2009) Myxinidin, a novel antimicrobial peptide from the epidermal mucus of hagfish, Myxine glutinosa L. Mar Biotechnol (NY) 11: 748–757. 19330556 10.1007/s10126-009-9189-y

[16] Easy RH, Ross NW (2010) Changes in Atlantic salmon Salmo salar mucus components following short- and long-term handling stress. Journal of Fish Biology 77: 1616–1631. 21078022 10.1111/j.1095-8649.2010.02796.x

[17] Tasumi S, Yang WJ, Usami T, Tsutsui S, Ohira T, et al (2004) Characteristics and primary structure of a galectin in the skin mucus of the Japanese eel, Anguilla japonica. Developmental and Comparative Immunology 28: 325–335. 14698218 10.1016/j.dci.2003.08.006

[18] Cole AM, Weis P, Diamond G (1997) Isolation and characterization of pleurocidin, an antimicrobial peptide in the skin secretions of winter flounder. Journal of Biological Chemistry 272: 12008–12013. 9115266 10.1074/jbc.272.18.12008

[19] Lauth X, Shike H, Burns JC, Westerman ME, Ostland VE, et al (2002) Discovery and characterization of two isoforms of moronecidin, a novel antimicrobial peptide from hybrid striped bass. Journal of Biological Chemistry 277: 5030–5039. 11739390 10.1074/jbc.M109173200

[20] Subramanian S, MacKinnon SL, Ross NW (2007) A comparative study on innate immune parameters in the epidermal mucus of various fish species. Comparative Biochemistry and Physiology B-Biochemistry & Molecular Biology 148: 256–263. 10.1016/j.cbpb.2007.06.003 17618153

[21] Al-Hassan JM, Thomson M, Criddle RS (1983) Accelerated wound healing by a preparation from skin of the Arabian Gulf catfish. Lancet 1: 1043–1044. 10.1016/s0140-6736(83)92665-x 6133079

[22] Zhang CX, Huang KX (2006) Mechanism of apoptosis induced by a polysaccharide, from the loach Misgurnus anguillicaudatus (MAP) in human hepatocellular carcinoma cells. Toxicology and Applied Pharmacology 210: 236–245. 15936790 10.1016/j.taap.2005.04.019

[23] Bureau of Fisheries MoA (2011) China Fisheries Statistical Yearbook. Beijing: China Agricultural Press.

[24] You L, Zhao M, Liu RH, Regenstein JM (2011) Antioxidant and antiproliferative activities of loach (Misgurnus anguillicaudatus) peptides prepared by papain digestion. Journal of Agricultural and Food Chemistry 59: 7948–7953. 21675717 10.1021/jf2016368

[25] Zhang CX, Huang KX (2005) Apoptosis induction on HL-60 cells of a novel polysaccharide from the mucus of the loach, Misgurnus anguillicaudatus. Journal of Ethnopharmacology 99: 385–390. 15935580 10.1016/j.jep.2005.02.033

[26] Qin CG, Ding Y, Huang KX, Xu HB (2008) Protective effect of Misgurnus anguillicaudatus polysaccharide on immunological liver injury in mice. International Immunopharmacology 8: 607–612. 18387502 10.1016/j.intimp.2007.12.015

[27] Park CB, Lee JH, Park IY, Kim MS, Kim SC (1997) A novel antimicrobial peptide from the loach, Misgurnus anguillicaudatus. FEBS Letters 411: 173–178. 9271200 10.1016/s0014-5793(97)00684-4

[28] Nam YK, Cho YS, Lee SY, Kim BS, Kim DS (2011) Molecular characterization of hepcidin gene from mud loach (Misgurnus mizolepis; Cypriniformes). Fish & Shellfish Immunology 31: 1251–1258. 21959039 10.1016/j.fsi.2011.09.007

[29] Dong XZ, Xu HB, Huang KX, Liou Q, Zhou J (2002) The preparation and characterization of an antimicrobial polypeptide from the loach, Misgurnus anguillicaudatus. Protein Expression and Purification 26: 235–242. 12406677 10.1016/s1046-5928(02)00514-4

[30] Aanes H, Winata CL, Lin CH, Chen JP, Srinivasan KG, et al (2011) Zebrafish mRNA sequencing deciphers novelties in transcriptome dynamics during maternal to zygotic transition. Genome Res 21: 1328–1338. 21555364 10.1101/gr.116012.110 PMC3149499

[31] Ji P, Liu G, Xu J, Wang X, Li J, et al (2012) Characterization of common carp transcriptome: sequencing, de novo assembly, annotation and comparative genomics. PLoS One 7: e35152. 22514716 10.1371/journal.pone.0035152 PMC3325976

[32] Fu B, He S (2012) Transcriptome analysis of silver carp (Hypophthalmichthys molitrix) by paired-end RNA sequencing. DNA Research 19: 131–142. 22279088 10.1093/dnares/dsr046 PMC3325077

[33] Xia JH, He XP, Bai ZY, Lin G, Yue GH (2011) Analysis of the Asian seabass transcriptome based on expressed sequence tags. DNA Research 18: 513–522. 22086997 10.1093/dnares/dsr036 PMC3223082

[34] Fraser BA, Weadick CJ, Janowitz I, Rodd FH, Hughes KA (2011) Sequencing and characterization of the guppy (Poecilia reticulata) transcriptome. BMC Genomics 12: 202. 21507250 10.1186/1471-2164-12-202 PMC3113783

[35] Hale MC, McCormick CR, Jackson JR, DeWoody JA (2009) Next-generation pyrosequencing of gonad transcriptomes in the polyploid lake sturgeon (Acipenser fulvescens): the relative merits of normalization and rarefaction in gene discovery. BMC Genomics 10. 10.1186/1471-2164-10-203 PMC2688523 19402907

[36] Coppe A, Pujolar JM, Maes GE, Larsen PF, Hansen MM, et al (2010) Sequencing, de novo annotation and analysis of the first Anguilla anguilla transcriptome: EeelBase opens new perspectives for the study of the critically endangered European eel. BMC Genomics 11: 635. 21080939 10.1186/1471-2164-11-635 PMC3012609

[37] Gao Z, Luo W, Liu H, Zeng C, Liu X, et al (2012) Transcriptome Analysis and SSR/SNP Markers Information of the Blunt Snout Bream (Megalobrama amblycephala). PLoS One 7: e42637. 22880060 10.1371/journal.pone.0042637 PMC3412804

[38] Schulz MH, Zerbino DR, Vingron M, Birney E (2012) Oases: robust de novo RNA-seq assembly across the dynamic range of expression levels. Bioinformatics 28: 1086–1092. 22368243 10.1093/bioinformatics/bts094 PMC3324515

[39] Grabherr MG, Haas BJ, Yassour M, Levin JZ, Thompson DA, et al (2011) Full-length transcriptome assembly from RNA-Seq data without a reference genome. Nature Biotechnology 29: 644–U130. 10.1038/nbt.1883 PMC3571712 21572440

[40] Surget-Groba Y, Montoya-Burgos JI (2010) Optimization of de novo transcriptome assembly from next-generation sequencing data. Genome Res 20: 1432–1440. 20693479 10.1101/gr.103846.109 PMC2945192

[41] Pertea G, Huang X, Liang F, Antonescu V, Sultana R, et al (2003) TIGR Gene Indices clustering tools (TGICL): a software system for fast clustering of large EST datasets. Bioinformatics 19: 651–652. 12651724 10.1093/bioinformatics/btg034

[42] Wang XW, Luan JB, Li JM, Bao YY, Zhang CX, et al (2010) De novo characterization of a whitefly transcriptome and analysis of its gene expression during development. BMC Genomics 11: 400. 20573269 10.1186/1471-2164-11-400 PMC2898760

[43] Li D, Deng Z, Qin B, Liu X, Men Z (2012) De novo assembly and characterization of bark transcriptome using Illumina sequencing and development of EST-SSR markers in rubber tree (Hevea brasiliensis Muell. Arg.). BMC Genomics 13: 192. 22607098 10.1186/1471-2164-13-192 PMC3431226

[44] Li B, Ruotti V, Stewart RM, Thomson JA, Dewey CN (2010) RNA-Seq gene expression estimation with read mapping uncertainty. Bioinformatics 26: 493–500. 20022975 10.1093/bioinformatics/btp692 PMC2820677

[45] Li B, Dewey CN (2011) RSEM: accurate transcript quantification from RNA-Seq data with or without a reference genome. BMC Bioinformatics 12. 10.1186/1471-2105-12-323 PMC3163565 21816040

[46] Hsiao CD, Ekker M, Tsai HJ (2003) Skin-specific expression of ictacalcin, a homolog of the S100 genes, during zebrafish embryogenesis. Developmental Dynamics 228: 745–750. 14648852 10.1002/dvdy.10411

[47] Conesa A, Gotz S, Garcia-Gomez JM, Terol J, Talon M, et al (2005) Blast2GO: a universal tool for annotation, visualization and analysis in functional genomics research. Bioinformatics 21: 3674–3676. 16081474 10.1093/bioinformatics/bti610

[48] Moriya Y, Itoh M, Okuda S, Yoshizawa AC, Kanehisa M (2007) KAAS: an automatic genome annotation and pathway reconstruction server. Nucleic Acids Res 35: W182–185. 17526522 10.1093/nar/gkm321 PMC1933193

[49] Burge SW, Daub J, Eberhardt R, Tate J, Barquist L, et al (2013) Rfam 11.0: 10 years of RNA families. Nucleic Acids Res 41: D226–232. 23125362 10.1093/nar/gks1005 PMC3531072

[50] Pereiro P, Balseiro P, Romero A, Dios S, Forn-Cuni G, et al (2012) High-Throughput Sequence Analysis of Turbot (Scophthalmus maximus) Transcriptome Using 454-Pyrosequencing for the Discovery of Antiviral Immune Genes. PLoS One 7. 10.1371/journal.pone.0035369 PMC3356354 22629298

[51] Chen J, Li C, Huang R, Du F, Liao L, et al (2012) Transcriptome analysis of head kidney in grass carp and discovery of immune-related genes. BMC Veterinary Research 8: 108. 22776770 10.1186/1746-6148-8-108 PMC3505460

[52] Strous GJ, Dekker J (1992) Mucin-type glycoproteins. Critical Reviews in Biochemistry and Molecular Biology 27: 57–92. 1727693 10.3109/10409239209082559

[53] Marel M, Adamek M, Gonzalez SF, Frost P, Rombout JH, et al (2012) Molecular cloning and expression of two beta-defensin and two mucin genes in common carp (Cyprinus carpio L.) and their up-regulation after beta-glucan feeding. Fish & Shellfish Immunology 32: 494–501. 22227003 10.1016/j.fsi.2011.12.008

[54] Brown GA, Wellings SR (1970) Electron microscopy of the skin of the teleost, Hippoglossoides elassodon. Zeitschrift fur Zellforschung und Mikroskopische Anatomie 103: 149–169. 5412825 10.1007/BF00337309

[55] Verdugo P (1990) Goblet cells secretion and mucogenesis. Annual Review of Physiology 52: 157–176. 10.1146/annurev.ph.52.030190.001105 2184755

[56] Goda Y (1997) SNAREs and regulated vesicle exocytosis. Proceedings of the National Academy of Sciences of the United States of America 94: 769–772. 9023331 10.1073/pnas.94.3.769 PMC33653

[57] Buchmann K (1999) Immune mechanisms in fish skin against monogeneans–a model. Folia Parasitologica 46: 1–9. 10408955

[58] Kasai K, Ishikawa T, Komata T, Fukuchi K, Chiba M, et al (2010) Novel L-amino acid oxidase with antibacterial activity against methicillin-resistant Staphylococcus aureus isolated from epidermal mucus of the flounder Platichthys stellatus. FEBS Journal 277: 453–465. 20015076 10.1111/j.1742-4658.2009.07497.x

[59] Wang Z, Fang B, Chen J, Zhang X, Luo Z, et al (2010) De novo assembly and characterization of root transcriptome using Illumina paired-end sequencing and development of cSSR markers in sweet potato (Ipomoea batatas). BMC Genomics 11: 726. 21182800 10.1186/1471-2164-11-726 PMC3016421

[60] Kanehisa M, Goto S, Kawashima S, Okuno Y, Hattori M (2004) The KEGG resource for deciphering the genome. Nucleic Acids Res 32: D277–280. 14681412 10.1093/nar/gkh063 PMC308797

[61] Pollard TD (2003) The cytoskeleton, cellular motility and the reductionist agenda. Nature 422: 741–745. 12700767 10.1038/nature01598

[62] Wozniak MA, Modzelewska K, Kwong L, Keely PJ (2004) Focal adhesion regulation of cell behavior. Biochimica et Biophysica Acta 1692: 103–119. 15246682 10.1016/j.bbamcr.2004.04.007

[63] Mu Y, Ding F, Cui P, Ao J, Hu S, et al (2010) Transcriptome and expression profiling analysis revealed changes of multiple signaling pathways involved in immunity in the large yellow croaker during Aeromonas hydrophila infection. BMC Genomics 11: 506. 20858287 10.1186/1471-2164-11-506 PMC2997002

[64] Hanisch FG (2001) O-glycosylation of the mucin type. Biological Chemistry 382: 143–149. 11308013 10.1515/BC.2001.022

[65] Tian E, Ten Hagen KG (2009) Recent insights into the biological roles of mucin-type O-glycosylation. Glycoconjugate Journal 26: 325–334. 18695988 10.1007/s10719-008-9162-4 PMC2656418

[66] Micallef G, Bickerdike R, Reiff C, Fernandes JM, Bowman AS, et al (2012) Exploring the Transcriptome of Atlantic Salmon (Salmo salar) Skin, a Major Defense Organ. Mar Biotechnol (NY) 14: 559–569. 22527268 10.1007/s10126-012-9447-2

[67] Hagen FK, Van Wuyckhuyse B, Tabak LA (1993) Purification, cloning, and expression of a bovine UDP-GalNAc: polypeptide N-acetyl-galactosaminyltransferase. Journal of Biological Chemistry 268: 18960–18965. 8360184

[68] Gerst JE (1999) SNAREs and SNARE regulators in membrane fusion and exocytosis. Cellular and Molecular Life Sciences 55: 707–734. 10379359 10.1007/s000180050328 PMC11146948

[69] Nagahama M, Orci L, Ravazzola M, Amherdt M, Lacomis L, et al (1996) A v-SNARE implicated in intra-Golgi transport. Journal of Cell Biology 133: 507–516. 8636227 10.1083/jcb.133.3.507 PMC2120813

[70] Wilson JM, Bunte RM, Carty AJ (2009) Evaluation of Rapid Cooling and Tricaine Methanesulfonate (MS222) as Methods of Euthanasia in Zebrafish (Danio rerio). Journal of the American Association for Laboratory Animal Science 48: 785–789. 19930828 PMC2786934

[71] Blessing JJ, Marshall JC, Balcombe SR (2010) Humane killing of fishes for scientific research: a comparison of two methods. Journal of Fish Biology 76: 2571–2577. 20557609 10.1111/j.1095-8649.2010.02633.x

[72] care Ccoa (2005) Guidelines on: the care and use of fish in research, teaching and testing.

[73] Schmieder R, Edwards R (2011) Quality control and preprocessing of metagenomic datasets. Bioinformatics 27: 863–864. 21278185 10.1093/bioinformatics/btr026 PMC3051327

[74] Zerbino DR, Birney E (2008) Velvet: Algorithms for de novo short read assembly using de Bruijn graphs. Genome Research 18: 821–829. 18349386 10.1101/gr.074492.107 PMC2336801

[75] Ye J, Fang L, Zheng HK, Zhang Y, Chen J, et al (2006) WEGO: a web tool for plotting GO annotations. Nucleic Acids Research 34: W293–W297. 16845012 10.1093/nar/gkl031 PMC1538768

