# Supplementary material for: De Novo Assembly of Mud Loach (Misgurnus anguillicaudatus) Skin Transcriptome to Identify Putative Genes Involved in Immunity and Epidermal Mucus Secretion
Source: PLoS One. 2013 Feb 20;8(2):e56998. doi: 10.1371/journal.pone.0056998 (PMC3577766; doi:10.1371/journal.pone.0056998)
Supplement: Figure S1 — RT-PCR results for selected unigenes. (PDF) [file pone.0056998.s001.pdf]

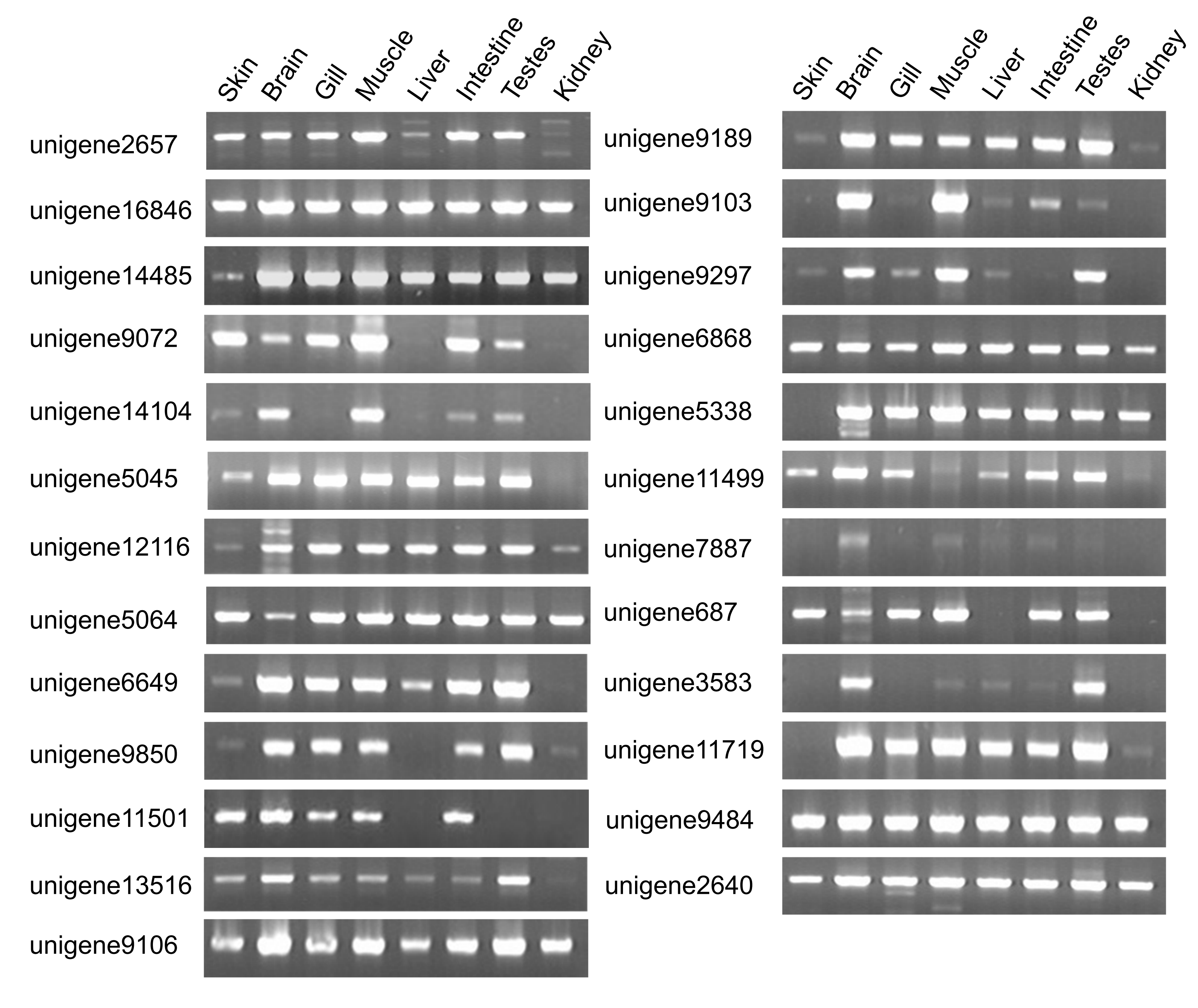

Skin
Brain
Gill
Muscle
Liver
Intestine
Testes
Kidney
unigene2657
unigene16846
unigene14485
unigene9072
unigene14104
unigene5045
unigene12116
unigene5064
unigene6649
unigene9850
unigene11501
unigene13516
unigene9106
unigene9189
unigene9103
unigene9297
unigene6868
unigene5338
unigene11499
unigene7887
unigene687
unigene3583
unigene11719
unigene9484
unigene2640
